# Supplementary material for: Impact of substrates and quantum effects on exciton line shapes of 2D semiconductors at room temperature
Source: Nanophotonics. 2023 Jun 20;12(16):3291–300. doi: 10.1515/nanoph-2023-0193 (PMC11501823; doi:10.1515/nanoph-2023-0193)
Supplement: Supplementary file 1 — Supplementary Material Details [file j_nanoph-2023-0193_suppl_001.pdf]

6 Supplemental Information

6.1 Ellipsometry data

The data file *Ellipsometry_data.txt* is available as Supplementary Data online and lists the refractive index $\tilde{n} = n + i\kappa$ measured with spectroscopic ellipsometry on a CVD-grown monolayer WS_2 on a fused silica substrate. The data is represented in Fig. 2. The sample is obtained commercially from 2Dsemiconductors (USA).

6.2 Quantum description of the reflection from WS_2 monolayer embedded in different dielectric environments

To calculate the reflection spectrum of the WS_2 monolayer embedded in different dielectric environments, we consider a three-layer dielectric stack, where the top, medium, and bottom layer correspond to the refractive index oil, the dielectric background of the WS_2 monolayer, and the fused silica substrate, respectively. Two planar dipole arrays are placed in the center of the medium layer ($z = 0$), representing A and B excitons in the WS_2 monolayer. The positive frequency part of the field operator at position z can be expressed as:

$$E(z,t) = E_0(z,t) + \frac{k_0^2}{\varepsilon_0} G(z) P_A(t) + \frac{k_0^2}{\varepsilon_0} G(z) P_B(t) \tag{3}$$

$E_0(z,t)$ represents the field distribution in the absence of exciton resonances, and $P_A(t) = \mu_A c_A(t)$ denotes the two-dimensional polarization induced by the A exciton resonance under a plane-wave illumination E_{inc}, where μ_A is the dipole moment of the exciton transition and $c_A(t)$ is the exciton annihilation operator. k_0 indicates the free-space wave number of the incident electric field and ε_0 is the permittivity of free space. We note that μ_A is fundamentally linked to the intrinsic radiative decay rate of excitons in vacuum γ_{rA} as:

$$\gamma_{rA} = \frac{k_0 \mu_A^2}{\varepsilon_0} \tag{4}$$

Identical relations for $P_B(t)$, μ_B, c_B, and γ_{rB} can be defined for the B exciton resonance. Meanwhile, $G(z)$ denotes the Green's function used to connect the polarization induced by exciton resonances to its scattered electric field, and thus can be further written as $G(z) = Ge^{ikz}$ when z is the position above the WS_2 monolayer. The total reflected electric field is therefore found to be:

$$E_r(z,t) = r_0(z) E_{inc} + \frac{k_0^2}{\varepsilon_0} G(z) \mu_A c_A(t) + \frac{k_0^2}{\varepsilon_0} G(z) \mu_B c_B(t) \tag{5}$$

$r_0(z) E_{inc} = r_0 E_{inc} e^{ikz}$ is the reflected electric field from the non-resonant background. If we ignore the interference between the A exciton and B exciton radiation as they are spectrally non-overlapping, the expectation value of the reflectance at z_0 from such a material stack is given by:

$$R = |r_0|^2 + \left(\frac{k_0^2 \mu_A}{\varepsilon_0}\right)^2 |G|^2 \frac{\langle c_A^\dagger c_A \rangle}{|E_{inc}|^2} + \left(\frac{k_0^2 \mu_B}{\varepsilon_0}\right)^2 |G|^2 \frac{\langle c_B^\dagger c_B \rangle}{|E_{inc}|^2} + 2\frac{k_0^2 \mu_A}{\varepsilon_0} \Re\left[r_0^* G \frac{\langle c_A \rangle}{E_{inc}}\right] + 2\frac{k_0^2 \mu_B}{\varepsilon_0} \Re\left[r_0^* G \frac{\langle c_B \rangle}{E_{inc}}\right] \tag{6}$$

The first term indicates the non-resonant background reflection in the absence of the exciton resonance, and the second and third term are the direct reflection from the A and B exciton radiation by self-interference. The last two terms, however, evaluate the interference between the reflection from the non-resonant background and that from the A and B exciton radiation.

Up to now, the analysis seems still fully classical. Nevertheless, the quantum description becomes necessary with the presence of the dephasing of the exciton polarization relative to the excitation field, as it

results in an exciton radiation contribution that is not mutually coherent with the background reflection, weakening the interference effects and thus affecting the exact line shape in the reflection spectra. For a more concise description, we focus on the A exciton here, but the conclusions hold for the B exciton as well. From Eq. 6, the weakened interference due to the dephasing should lead to an inequality:

$$\langle c_A^\dagger c_A \rangle > |\langle c_A \rangle|^2, \tag{7}$$

a relation that we will prove from the quantum mechanical description as discussed below. Specifically, the exciton dynamics can be described by two master equations:

$$\frac{d}{dt}\langle c_A \rangle = i(\delta_A - \Delta_A + i\tilde{\gamma_A})\langle c_A \rangle + i\mu_A E_0, \tag{8}$$

$$\frac{d}{dt}\langle c_A^\dagger c_A \rangle = -\gamma_A \langle c_A^\dagger c_A \rangle + i\mu_A E_0 \langle c_A^\dagger \rangle - i\mu_A E_0^\dagger \langle c_A \rangle. \tag{9}$$

In these equations, $\delta_A = \omega - \omega_A$ is the detuning of the frequency of the incident light ω with respect to the exciton resonance frequency ω_A. $\Delta_A = 0.5\gamma_{rA} k_0 \Re[G(0)]$ is the shift of the exciton resonant frequency modified by the local photonic environment (i.e., the Lamb shift). $\tilde{\gamma_A} = \gamma_{dA} + \frac{1}{2}\gamma_A = \gamma_{dA} + \frac{1}{2}\left(\gamma_{nrA} + 2\gamma_{rA} k_0 \Im[G(0)]\right)$ represents the total decay rate of excitons, being the sum of the pure dephasing rate γ_{dA} and the population decay rate γ_A. The non-radiative decay rate is represented by γ_{nrA}, and $2\gamma_{rA} k_0 \Im[G(0)]$ is the external decay rate modified by the local photonic environment as well (i.e., the Purcell effect). Finally, $E_0 = E_0(z = 0)$ for simplicity.

At steady state, the expectation value of the exciton annihilation $\langle c_A \rangle$ and exciton population $\langle c_A^\dagger c_A \rangle$ are found to be:

$$\langle c_A \rangle = \frac{-i\mu_A E_0}{i(\delta_A - \Delta_A + i\tilde{\gamma_A})}, \tag{10}$$

$$\langle c_A^\dagger c_A \rangle = \frac{2\mu_A^2 |E_0|^2 \tilde{\gamma_A}}{\gamma_A \left((\delta_A - \Delta_A)^2 + \tilde{\gamma_A}^2\right)}. \tag{11}$$

Therefore, we find that the weakened interference is quantitatively related to the presence of the dephasing, and vanishes when $\gamma_{dA} = 0$:

$$\langle c_A^\dagger c_A \rangle = \frac{2\gamma_{dA} + \gamma_A}{\gamma_A} |\langle c_A \rangle|^2 > |\langle c_A \rangle|^2. \tag{12}$$

By plugging the above equations into Eq. 6, we get:

$$\begin{aligned} R = |r_0|^2 + k_0^2 |G|^2 \frac{2\tilde{\gamma_A}\gamma_{rA}^2 |E_0|^2}{\gamma_A \left((\delta_A - \Delta_A)^2 + \tilde{\gamma_A}^2\right) |E_{inc}|^2} \\ + k_0^2 |G|^2 \frac{2\tilde{\gamma_B}\gamma_{rB}^2 |E_0|^2}{\gamma_B \left((\delta_B - \Delta_B)^2 + \tilde{\gamma_B}^2\right) |E_{inc}|^2} \\ + 2\gamma_{rA} k_0 \Re\left[r_0^* G \frac{-iE_0}{i\left(\delta_A - \Delta_A + i\tilde{\gamma_A}\right) E_{inc})}\right] \\ + 2\gamma_{rB} k_0 \Re\left[r_0^* G \frac{-iE_0}{i\left(\delta_B - \Delta_B + i\tilde{\gamma_B}\right) E_{inc})}\right] \end{aligned} \tag{13}$$

We note that such an equation can be calculated analytically with a transfer-matrix approach given a set of exciton decay rates $\left(\gamma_{rA(B)}, \gamma_{nrA(B)}, \gamma_{dA(B)}\right)$. Conversely, with the measured reflection spectra in hand, we can apply a mean-square error minimization fitting routine to extract the most probable intrinsic radiative decay, non-radiative decay, and pure dephasing rates of the A and B excitons in the characterized WS_2 monolayer, as shown in Fig. 4.
